# Optimization and Deployment of Real-Time On-Orbit Intelligent Interpretation Algorithms for Spaceborne Remote Sensing

**DOI:** 10.3390/s26144377

**Published:** 2026-07-10

**Authors:** Cankai Li, Haiming Jiang, Yanwei Li, Hongbo Xie, Yipeng Wang, Yongxiang Fan

**Affiliations:** 1 School of Mechanical and Electrical Engineering, Guangdong University of Technology, Guangzhou 510006, China; licankai@mails.gdut.edu.cn; 2 The Advanced Optoelectronic Intelligent Equipment Research Group, Ji Hua Laboratory, Foshan 528200, China; xiehb@jihualab.ac.cn (H.X.); wangypeng@jihualab.ac.cn (Y.W.); fanyx@jihualab.ac.cn (Y.F.)

**Keywords:** FPGA, SWaP, YOLOv8, CNN, object detection

## Abstract

Orbital remote sensing platforms increasingly rely on CNN-based object detection for real-time situational awareness. However, deploying these models on spaceborne edge devices is challenging because of stringent Size, Weight, and Power (SWaP) constraints. In addition, the branch-and-merge topology of conventional single-stage detectors increases on-chip memory usage and introduces pipeline stalls, limiting efficient FPGA implementation. To address these challenges, we proposed RS-YOLO, an object detection algorithm developed through a hardware–software co-design approach. Structural re-parameterization converts heterogeneous branches into a sequential stream of padding-free convolutions, producing a deterministic dataflow and reducing per-state combinational control complexity and data-path multiplexing overhead. To mitigate the high-entropy concentration at the center of the re-parameterized kernels, we further introduce a spatial heterogeneous quantization (SHQ) engine. The SHQ engine assigns 16-bit precision to the central coefficients while preserving vectorized 8-bit computation for peripheral elements, reducing quantization errors for small targets with minimal hardware overhead. Experimental results on the Xilinx Zynq-7020 platform show that the proposed system consumes only 2.24 W while achieving a mean Average Precision (mAP) of 0.887 on the NWPU VHR-10 dataset, representing a 1.4% decrease compared with the FP32 baseline. The system also achieves an energy efficiency of 15.19 GOPS/W, demonstrating an effective balance between hardware efficiency and detection performance for resource-constrained edge platforms such as micro-satellite payloads.

## 1. Introduction

Driven by advances in aerospace remote sensing and high-resolution Earth observation, satellite-borne optical sensors can acquire large volumes of wide-swath imagery [1]. However, the traditional transmit-then-process paradigm is constrained by limited downlink bandwidth and communication latency, making it difficult to satisfy the real-time requirements of time-critical applications such as disaster monitoring and early warning. As a result, on-orbit intelligent interpretation has emerged as an important direction in remote sensing by moving computation from ground stations to satellite payloads [2,3]. Nevertheless, deploying deep learning models on satellite platforms remains challenging because their computational demands conflict with the stringent Size, Weight, and Power (SWaP) constraints of spaceborne systems [4].

Among deep learning-based object detection methods, convolutional neural networks (CNNs) have consistently outperformed traditional hand-crafted feature extractors in feature representation [5,6]. For on-orbit applications, however, detection models must balance computational efficiency and detection accuracy. Compared with two-stage detectors such as Faster R-CNN [7], the YOLO (You Only Look Once) family formulates object localization and classification as a unified regression task, substantially reducing inference latency [8]. Its end-to-end architecture also reduces intermediate memory access and offers high throughput when processing large-scale remote sensing imagery [9]. These advantages make YOLO well suited for on-orbit intelligent processing under limited computing resources [10,11,12].

Although GPU platforms such as NVIDIA Jetson provide a flexible development environment, their high power consumption and limited radiation tolerance restrict their use in spaceborne applications. Field-Programmable Gate Arrays (FPGAs), in contrast, offer high energy efficiency, deterministic latency, and inherent parallelism, making them well suited for satellite payloads. However, implementing modern YOLO networks on FPGAs remains challenging. Traditional register-transfer level (RTL) design requires long development cycles and makes efficient mapping of complex network topologies difficult [13]. High-Level Synthesis (HLS) simplifies the development process, but straightforward HLS translation without operator reorganization or parallelism optimization often results in poor DSP utilization and insufficient throughput for remote sensing applications [14]. In recent years, researchers have proposed various hardware design architectures to address these issues. For instance, Zhang et al. [15] introduced an (ARM+FPGA) reconfigurable accelerator optimized via dynamic fixed-point quantization for YOLOv2 variants, successfully cutting down off-chip memory access frequencies. Babu et al. [16] enhanced external memory bandwidth utilization through a coordinated orchestration of loop pipelining and data tiling techniques tailored for YOLOv4 inference. Fang et al. [17] engineered a hybrid overlapped acceleration architecture leveraging intra-layer fusion and double-buffering mechanisms to compress YOLOv5s latency, while Yan et al. [18] explored extreme ultra-low 4-bit quantization accompanied by unified DSP packing strategies to scale down hardware resource consumption.

Despite these advances, a gap remains between lightweight network design and efficient FPGA implementation for spaceborne remote sensing [19]. Existing lightweight networks typically reduce model complexity by introducing multi-branch structures, including cross-layer connections, split-and-merge operations, and residual paths [20]. Although these designs are effective on GPUs, they complicate FPGA implementation. For example, the branch-and-merge operations in the C2f module of YOLOv8 require intermediate feature maps to be buffered in on-chip Block RAM (BRAM) until multiple branches are synchronized [21,22]. In addition, branch-dependent control logic introduces data-path multiplexing and synchronization overhead, resulting in pipeline stalls and increased per-state combinational control complexity in the finite-state machine (FSM) [23].

Quantization presents another challenge. Most post-training quantization (PTQ) methods apply a uniform bit width to an entire layer or tensor, which cannot fully exploit the arithmetic capability of FPGA DSP resources [24]. More importantly, uniform quantization often leads to substantial accuracy degradation when processing the high-entropy features commonly found in remote sensing images [25]. These limitations indicate that improving spaceborne edge inference requires not only lightweight network design but also coordinated optimization of network architecture and hardware implementation.

To address these challenges, we propose an FPGA-oriented hardware–software co-design framework. The main contributions of this work are summarized as follows:

- Structural isomorphism through structural re-parameterization. The proposed RepStraight module transforms the multi-branch topology of the baseline network into a sequential stream of padding-free 3 × 3 convolutions before deployment. This structural simplification eliminates branch synchronization, reduces control complexity, and establishes a deterministic single-path dataflow for efficient FPGA implementation.
- Spatial heterogeneous quantization. The proposed SHQ engine addresses the spatial energy concentration introduced by structural re-parameterization. By assigning 16-bit precision to the central coefficients while preserving vectorized 8-bit computation for peripheral coefficients, the proposed quantization strategy preserves critical feature information without increasing the global bit width.
- Hardware–software co-design for FPGA deployment. The proposed hardware–software co-design framework integrates network optimization with FPGA implementation to improve execution efficiency on resource-constrained platforms. Experimental results on the Xilinx Zynq-7020 demonstrate that the proposed system operates within a total power budget of 2.24 W while maintaining reliable detection performance for dim and small targets.

## 2. Materials and Methods

### 2.1. Algorithm Improvement

YOLOv8n is the lightweight variant of the YOLO series and is implemented in PyTorch 2.4.1. As a typical single-stage detector, it formulates object detection as a regression problem by directly predicting bounding box coordinates, class probabilities, and confidence scores [26]. The YOLOv8 family includes five variants—YOLOv8n, YOLOv8s, YOLOv8m, YOLOv8l, and YOLOv8x—which share the same network architecture but differ in depth and width scaling. Among them, YOLOv8n has the fewest parameters and the lowest inference latency, making it well suited for deployment on resource-constrained edge platforms [27,28].

**Figure 1:**
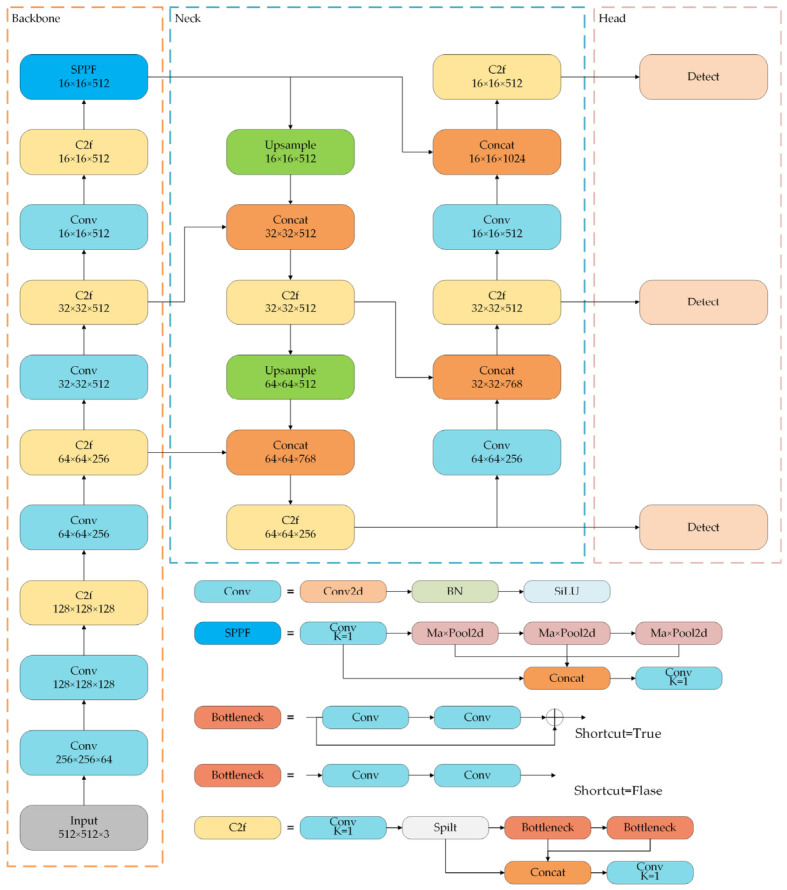
The architecture of YOLOv8n.

As shown in Figure 1, YOLOv8 consists of three components: the backbone, the neck, and the head. The backbone extracts multi-scale features from the input image and comprises Conv blocks, C2f modules, and a Spatial Pyramid Pooling-Fast (SPPF) module. Each Conv block consists of a convolution layer, a batch normalization (BN) layer, and a Sigmoid Linear Unit (SiLU) activation function [29]. The C2f module employs multi-branch connections to improve feature reuse and gradient propagation, while the SPPF module serializes pooling operations to enlarge the receptive field with low computational overhead.

The neck combines multi-scale features using a Feature Pyramid Network (FPN) and a Path Aggregation Network (PAN) before passing them to the detection head. The head predicts object classes and bounding box coordinates. This architecture reduces computational complexity while maintaining competitive detection accuracy, making YOLOv8 suitable for intelligent interpretation on resource-constrained edge platforms.

#### 2.1.1. RepStraight

Although single-stage detectors exhibit strong feature representation capabilities, their typical multi-branch architectures—particularly the cross-layer split-and-merge topologies instantiated by standard C2f blocks—impose severe constraints on spaceborne synchronous scheduling. In hardware execution contexts, standard C2f blocks require half of the intermediate channel features to be buffered inside on-chip memory to await multi-path data synchronization [30]. This topological heterogeneity increases the on-chip memory footprint because intermediate feature maps from parallel branches must be buffered and synchronized before aggregation. In addition, the coexistence of heterogeneous convolution operators, particularly the 1 × 1 and 3 × 3 convolutions in the standard C2f block, requires dynamic branch-selection control signals and corresponding data-path multiplexers. These factors increase both the per-state combinational routing complexity and the inter-branch synchronization overhead of the hardware control logic.

**Figure 2:**
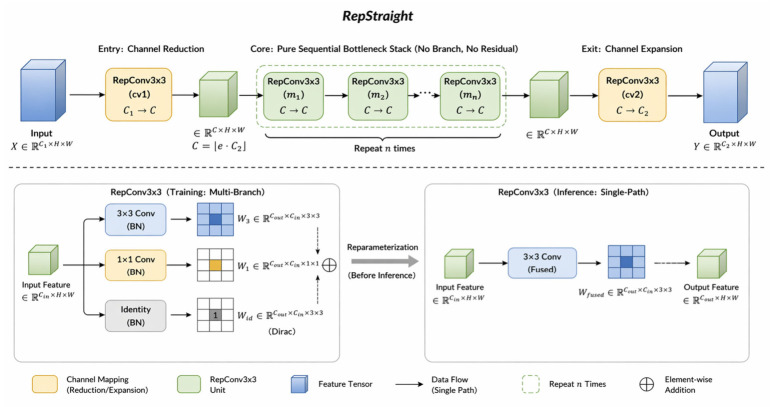
The structure diagram of RepStraight.

To neutralize these synchronous data movement bottlenecks under stringent SWaP constraints, this paper introduces the RepStraight module as a structural simplification paradigm, as illustrated in Figure 2. The core idea of RepStraight is to shift the design objective from GPU-oriented lightweight architectures to hardware-friendly structural isomorphism for FPGA implementation. Through structural re-parameterization, the irregular computation graph is transformed before deployment into a sequential single-path pipeline, thereby decoupling dataflow dependencies between heterogeneous branches. Let $X\in R^{C_{1}\times H\times W}$ and $Y\in R^{C_{2}\times H\times W}$ denote the input and output tensors, respectively. The inference-state sequential transformation flow within the RepStraight block is formally expressed as:

(1) $$Y=\Phi_{\mathrm{RepStraight}}\left(X\right)=K_{\mathrm{cv}2}*\sigma \left(K_{m,n}*\ldots \sigma \left(K_{m,1}*\sigma \left(K_{\mathrm{cv}1}*X\right)\right)\ldots \right)$$

where ∗ represents the two-dimensional spatial convolution operator, σ(⋅) denotes the non-linear activation function, and $K\in R^{C_{\mathrm{out}}\times C_{\mathrm{in}}\times 3\times 3}$ represents the structurally unified, padding-free re-parameterized 3×3 convolutional kernels.

Unlike conventional structural re-parameterization methods such as RepVGG [30] and related YOLO variants, which are primarily designed to reduce inference-time convolution redundancy and optimize execution efficiency on GPU platforms, their optimization targets remain focused on arithmetic fusion while largely overlooking hardware-level execution constraints such as routing complexity, control-flow divergence, and memory synchronization. In contrast, the proposed RepStraight module is explicitly designed for FPGA-oriented deployment, where deterministic dataflow and control-path simplicity are critical for achieving high resource utilization under stringent SWaP constraints.

RepStraight enforces operator-level uniformity not only at the convolutional arithmetic level but also at the execution graph level, thereby eliminating heterogeneous branch-selection logic and inter-branch synchronization that typically arise from mixed 1 × 1 and 3 × 3 convolution structures. As a result, the computation graph is transformed into a strictly sequential execution pipeline that is naturally compatible with systolic-array-based FPGA architectures. This design differs from prior re-parameterization approaches, which primarily focus on weight fusion but do not explicitly address hardware scheduling and control-path complexity.

During the training phase, the baseline computational core unrolls into three heterogeneous parallel forwarding paths: a regular spatial convolution, an auxiliary channel-squeezing convolution, and an identity shortcut. To eliminate runtime routing overhead, the algorithmic weights of these separate operators are analytically fused into a singular weight tensor prior to execution mapping. Let $W_{3\times 3}\in R^{C_{\mathrm{out}}\times C_{\mathrm{in}}\times 3\times 3}$ and $W_{1\times 1}\in R^{C_{\mathrm{out}}\times C_{\mathrm{in}}\times 1\times 1}$ represent the weight tensors derived after absorbing the BN layers, respectively. The spatial coefficients of the consolidated 3×3 operator, denoted as $W_{\mathrm{fused}}\in R^{C_{\mathrm{out}}\times C_{\mathrm{in}}\times 3\times 3}$, are rigorously computed via linear addition:

(2) $$W_{\mathrm{fused}}\left(c_{\mathrm{out}},c_{\mathrm{in}},p,q\right)=W_{3\times 3}\left(c_{\mathrm{out}},c_{\mathrm{in}},p,q\right)+T\left(W_{1\times 1}\right)\left(c_{\mathrm{out}},c_{\mathrm{in}},p,q\right)+\;W_{\mathrm{Dirac}}\left(c_{\mathrm{out}},c_{\mathrm{in}},p,q\right)$$

where $c_{\mathrm{out}}$ and $c_{\mathrm{in}}$ denote the output and input channel indices, and p,q∈{−1,0,1} represent the spatial offsets relative to the kernel’s geometric center. The spatial expansion operator T(⋅) maps the 1×1 kernel coefficients onto the central coefficients, while $W_{\mathrm{Dirac}}$ embodies the discrete unit impulse response tensor. These operators are mathematically governed by the Kronecker delta function δ(⋅,⋅) as follows:

(3) $$T\left(W_{1\times 1}\right)\left(c_{\mathrm{out}},c_{\mathrm{in}},p,q\right)=W_{1\times 1}\left(c_{\mathrm{out}},c_{\mathrm{in}},0,0\right)\cdot \delta \left(p,0\right)\cdot \delta \left(q,0\right)$$

(4) $$W_{\mathrm{Dirac}}\left(c_{\mathrm{out}},c_{\mathrm{in}},p,q\right)=\delta \left(c_{\mathrm{out}},c_{\mathrm{in}}\right)\cdot \delta \left(p,0\right)\cdot \delta \left(q,0\right)$$

By transforming the dynamic multi-branch training topology into a mathematically equivalent single-path 3 × 3 convolution during inference, the proposed RepStraight module provides two architectural advantages for FPGA implementation.

Deterministic Control-Path Simplification: The standard C2f module requires hardware-level switching between 1 × 1 projection and 3 × 3 convolution branches, introducing branch-selection multiplexers and conditional control signals into the FSM. RepStraight removes these intra-layer branch dependencies by re-parameterizing the heterogeneous computation graph into a single fixed-topology 3 × 3 convolution. As a result, hardware execution changes from a multi-mode scheduling process, in which the FSM must coordinate different execution paths and synchronize their partial results, to a deterministic sequential execution stream. Although the overall scheduling framework preserves the original state-transition structure, the combinational logic within each state is simplified because branch-selection multiplexers and inter-branch synchronization are eliminated. Consequently, the per-state control logic is reduced to a deterministic feed-forward routing pattern.

Memory Footprint Decoupling: In conventional multi-branch modules, intermediate feature maps generated by parallel branches must be buffered and synchronized before element-wise aggregation, introducing implicit storage and synchronization requirements for on-chip memory. By collapsing the parallel branches into a single convolution path, RepStraight removes the need for intermediate feature buffering within each layer. The output feature map is generated directly through a single sliding-window convolution. Consequently, the memory footprint of each layer becomes independent of the branch multiplicity of the training-time topology, allowing more BRAM resources to be allocated to feature-map tiling or weight caching.

At the hardware architecture level, these characteristics naturally support a systolic-array execution model. The fixed 3 × 3 kernel geometry and uniform stride pattern allow the convolution sequence to be mapped to a deterministic line-scanning schedule without runtime routing or pipeline reconfiguration. Consequently, the control flow is simplified from a conditional branch graph to a linear counter-driven address sequence, reducing pipeline stalls and improving the utilization of the underlying multiply–accumulate (MAC) units during continuous feature-map streaming.

#### 2.1.2. RS-YOLO

Building upon the structural simplification introduced by the RepStraight module, the overall architecture of RS-YOLO is designed to facilitate efficient FPGA implementation. The network adopts a multi-level topological regularization strategy to balance deterministic hardware execution with intelligent interpretation performance.

To prevent the structural and terminological ambiguities prevalent in conventional lightweight networks, the topological abstraction of RS-YOLO is rigorously partitioned into two functional dimensions:

- Intra-layer Micro-structural Isomorphism: Within each computational block, fine-grained operator heterogeneity—including the parallel branches, channel-splitting operations, and short-range residual connections in the original C2f module—is eliminated through the structural re-parameterization described in Section 2.1.1. Consequently, the backbone and local feature extraction modules are transformed into a sequential stream of homogeneous 3 × 3 convolution kernels. This structural regularization simplifies hardware implementation by removing dynamic data dependencies and establishing deterministic execution behavior.
- Macro-structural Scale Alignment: At the network level, RS-YOLO preserves the FPN to maintain multi-scale feature representations required for satellite remote sensing. To improve the detection of dim and small targets in wide-swath imagery, an additional high-resolution P2 feature branch is introduced. Unlike the fine-grained branching within the original C2f module, these feature concatenation and upsampling operations are performed only at coarse-grained stage boundaries, where synchronization naturally occurs between network stages.

**Figure 3:**
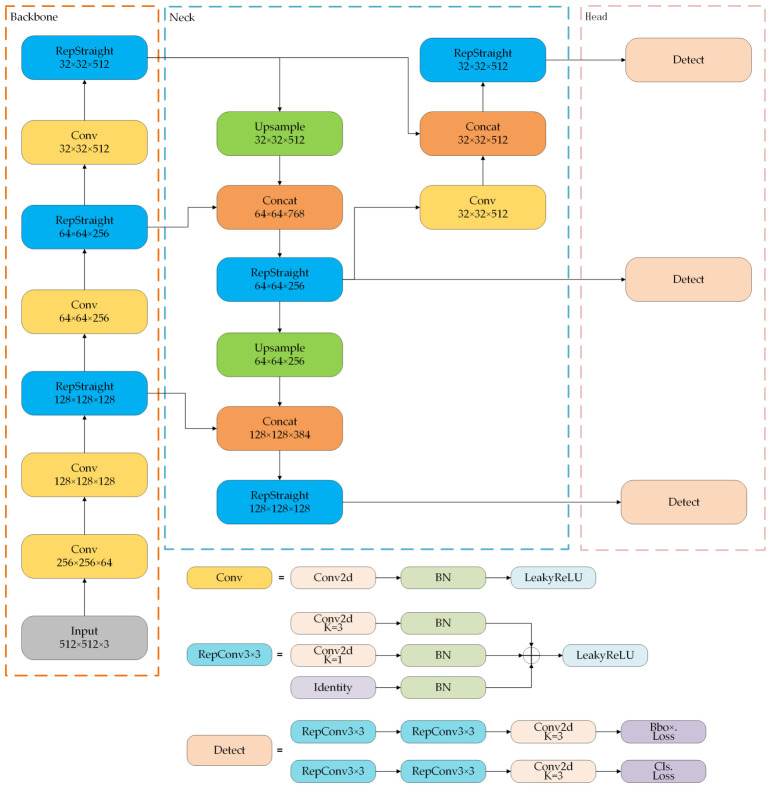
The architecture of RS-YOLO.

As illustrated in Figure 3, the resulting computation graph forms a deterministic dataflow. By separating fine-grained operator heterogeneity from coarse-grained multi-scale feature aggregation, RS-YOLO reduces runtime branch synchronization and control complexity while preserving multi-scale feature fusion. This architectural organization enables efficient hardware resource utilization and provides a suitable structural basis for the subsequent hardware–software co-design framework.

### 2.2. Information-Entropy Adaptive Quantization Strategy

As derived from the structural re-parameterization described in Section 2.1.1, the central coefficient of the fused 3 × 3 convolution kernel before inference, denoted as $W_{\mathrm{fused}}\left(c_{\mathrm{out}},c_{\mathrm{in}},0,0\right)$, accumulates the mathematical contributions of the original 3 × 3 convolution, the auxiliary 1 × 1 convolution, and the identity branch. Consequently, structural re-parameterization introduces a non-uniform spatial energy distribution within the fused convolution kernel.

**Figure 4:**
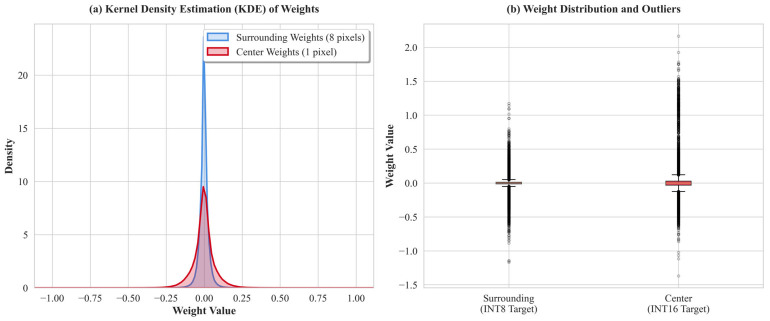
Weight distribution analysis.

From an information-theoretic perspective, the computational contributions of both the auxiliary 1 × 1 branch and the identity mapping are accumulated at the kernel center. As a result, the dynamic range of the central coefficient becomes substantially larger than that of the eight peripheral coefficients within the spatial domain Ω={(p,q)∣p,q∈{−1,0,1}∖{(0,0)}}. Consequently, the central coefficient exhibits higher information entropy and a heavier-tailed distribution than the surrounding coefficients. When conventional layer-wise uniform 8-bit post-training quantization (PTQ) is applied, the quantization scale $\Delta =\frac{\max-min}{2^{8}-1}$ must accommodate the enlarged dynamic range of the central coefficient. This enlarged quantization interval reduces the effective representation precision of the low-energy peripheral coefficients, causing subtle feature responses to be quantized to zero and degrading the representation of dim and small targets, as illustrated in Figure 4.

To address this conflict between dynamic range and quantization resolution without increasing the global bit width, the proposed spatial heterogeneous quantization (SHQ) engine separates the central coefficient from the peripheral coefficients during quantization. As illustrated by the coefficient distribution analysis, the central high-entropy coefficient $W_{\mathrm{fused}}\left(c_{\mathrm{out}},c_{\mathrm{in}},0,0\right)$ assigned an independent 16-bit multiplication path, whereas the remaining low-entropy coefficients within the spatial domain Ω are processed using a vectorized 8-bit computation pipeline. By partitioning the convolution kernel into spatial regions with different entropy characteristics, the SHQ engine reduces the quantization error of dim and small targets while introducing only a small hardware overhead, providing an appropriate numerical basis for the subsequent systolic-array accelerator.

### 2.3. Spatial Heterogeneous Quantization Engine

**Figure 5:**
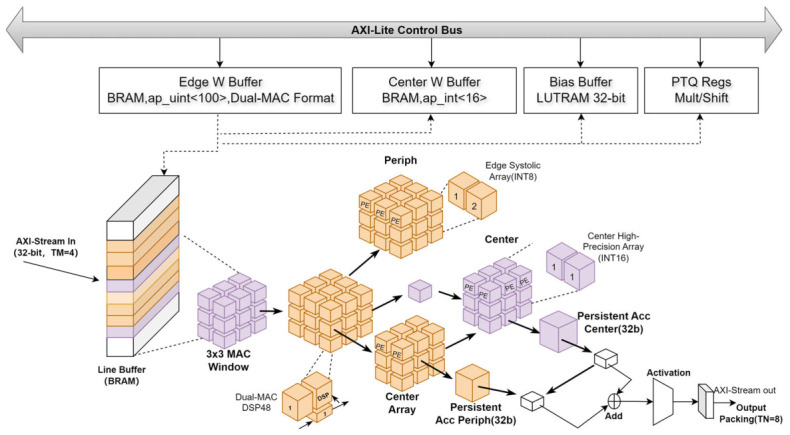
SHQ Computing Engine.

To implement the information-entropy adaptive quantization strategy described in Section 2.2, a dedicated SHQ operator IP core is developed. As illustrated in Figure 5, the hardware compute engine is tailored to the non-uniform spatial coefficient distribution of RS-YOLO. It performs heterogeneous bit-width computation directly in hardware, enabling high inference throughput while preserving numerical precision on the resource-constrained Xilinx Zynq-7020 platform.

#### 2.3.1. Neighborhood INT8 Computing Flow

For the low-entropy coefficients located in the peripheral spatial domain Ω, the processing element (PE) array employs a vectorized INT8 packing scheme to improve the computational density of the Xilinx DSP48E primitives. Each DSP48E slice provides an asymmetric 25 × 18-bit signed multiplier. This hardware characteristic allows two independent 8-bit peripheral coefficients, denoted as $w_{0}$ and $w_{1}$, to be packed into a single 25-bit weight word, ($W_{\mathrm{pack}}$).

However, directly packing two signed two’s-complement integers introduces sign-extension interference that affects the higher-order bits of the packed operand. To eliminate this numerical interference without introducing additional runtime control logic, an algebraic sign-bit pre-compensation strategy is adopted. The packing operation is defined as:

(5) $$W_{\mathrm{pack}}=\{\begin{array}{cc} \left(w_{1}\cdot 2^{16}\right)+w_{0}, & w_{0}\geq 0\\ \left(\left(w_{1}-1\right)\cdot 2^{16}\right)+\left(w_{0}+2^{16}\right), & w_{0}<0 \end{array}$$

The packed weight is then mapped to the 25-bit input port of the DSP multiplier and multiplied by the shared 8-bit feature activation (A). A single multiplication therefore generates the partial products of two peripheral coefficients within one clock cycle. This packing strategy doubles the computational utilization of each DSP48E slice. Combined with the ap_uint<100> wide-word weight storage format, it also reduces routing congestion in resource-constrained FPGA devices.

#### 2.3.2. Central Coefficient 16-Bit Computing Flow

To preserve the high-entropy structural information concentrated at the center of the re-parameterized kernel, a dedicated 16-bit multiplication path is implemented in parallel with the peripheral INT8 processing array. This independent computation path preserves the dynamic range of the central coefficient throughout the convolution process.

Because the peripheral INT8 array and the central INT16 path adopt different quantization scales, a dual-path dequantization architecture is introduced at the output stage of the IP core. The final convolution output, denoted by $Y_{\mathrm{out}}$ is obtained through cross-scale numerical fusion:

(6) $$Y_{\mathrm{out}}=\sigma \left(\sum_{i=0}^{7}P_{\mathrm{periph},i}\cdot \frac{M_{\mathrm{periph}}}{2^{n_{\mathrm{periph}}}}+P_{\mathrm{center}}\cdot \frac{M_{\mathrm{center}}}{2^{n_{\mathrm{center}}}}+B\right)$$

where $P_{\mathrm{periph},i}$ and $P_{\mathrm{center}}$ denote the accumulated fixed-point outputs of the peripheral systolic array and the central high-precision computation path, respectively. (M) and (*n*) represent the dequantization multipliers and the corresponding scaling shift parameters, while (B) denotes the hardware bias term. To avoid introducing additional latency into the accumulation pipeline, heterogeneous bit-width alignment is deferred until the final dequantization stage. As a result, the high-frequency accumulation loop is isolated from bit-width conversion and scaling operations on the critical path. This design enables the heterogeneous computing engine to operate with an initiation interval (II) of 1 while reducing logic resource consumption and maintaining reliable detection performance for dim and small targets.

### 2.4. Hardware–Software Co-Scheduling

To improve hardware resource utilization and reduce inter-layer communication latency, the proposed framework adopts a hierarchical asynchronous hardware–software co-design strategy. During system initialization, the Processing System (PS) transfers a global network profile vector, denoted as $\Psi_{\mathrm{global}}$, to the Programmable Logic (PL) through the AXI4-Lite control interface. This vector contains the static configuration parameters of the network:

(7) $$\Psi_{\mathrm{global}}=\left\{N_{\mathrm{depth}},A_{\mathrm{base}},T_{\mathrm{act}}\right\}$$

where $N_{\mathrm{depth}}$ denotes the total number of computational layers, $A_{\mathrm{base}}$ specifies the base address of the memory space, and $T_{\mathrm{act}}$ stores the precomputed activation thresholds. Together, these parameters define the static operating configuration of the hardware accelerator.

During initialization, the system enters a layer-by-layer execution mode. During runtime, the software driver updates only the descriptor of the active layer, denoted by $\Phi_{\mathrm{layer},i}$, while the remaining configuration remains unchanged. The complete configuration set is expressed as:

(8) $$\Gamma_{\mathrm{total}}=\left\{\Psi_{\mathrm{global}},\Phi_{\mathrm{layer},1},\Phi_{\mathrm{layer},2},\ldots ,\Phi_{\mathrm{layer},N}\right\}$$

(9) $$\mathrm{with}\;\Phi_{\mathrm{layer},i}=\left\{W_{i},H_{i},S_{i},C_{\mathrm{in},i},C_{\mathrm{out},i},M_{\mathrm{periph},i},n_{\mathrm{periph},i},M_{\mathrm{center},i},n_{\mathrm{center},i}\right\}$$

where $W_{i}$, $H_{i}$, and $S_{i}$ denote the spatial dimensions and stride of the feature map, $C_{\mathrm{in},i}$ and $C_{\mathrm{out},i}$ represent the input and output channel numbers, and (M) and (*n*) are the SHQ dequantization parameters defined in Section 2.3.2.

This hierarchical configuration mechanism enables the PL accelerator to operate autonomously after receiving the global network configuration. During inference, only a small set of layer-specific parameters is updated, reducing runtime communication overhead between the PS and PL. Consequently, the deterministic operator sequence can execute continuously with high processing-array utilization while minimizing host-side control overhead.

#### 2.4.1. Cache Coherence Protocol

To support the high-throughput processing requirements of wide-swath remote sensing imagery, the proposed architecture transfers weights and feature maps through two independent AXI4-Stream channels. Because both streams access external memory through the High-Performance (HP) ports of the Zynq platform, cache inconsistency may occur between the PS cache hierarchy and the asynchronous DMA engines in the PL. Instead of introducing hardware cache-coherence circuitry, the proposed framework adopts an explicit software-managed cache coherence protocol.

The memory consistency between the heterogeneous domains is governed by the following operational sequence. First, prior to triggering PL execution, the input tensor data undergoes a cache flush operation to ensure that the PL fetches the updated input imagery:

(10) $$D_{\mathrm{in}\text{\_}\mathrm{safe}}={\mathrm{Flush}}_{\mathrm{PS}}\left(D_{\mathrm{input}}\right)$$

where ${\mathrm{Flush}}_{\mathrm{PS}}\left(\cdot \right)$ synchronizes dirty data from the processor L1/L2 cache lines back into the external DDR memory.

Subsequently, the spatial computing engine on the PL side consumes this validated safe data domain, executing the isomorphic convolution operator sequence to generate the intermediate feature maps:

(11) $$D_{\mathrm{PL}\text{\_}\mathrm{out}}=\Omega_{\mathrm{PL}}\left(D_{\mathrm{in}\text{\_}\mathrm{safe}}\right)$$

Finally, to completely prevent data incoherence during the multi-stage inter-layer handover, the driver applies a localized invalidate operation to the target destination memory segment before the PS reads the output:

(12) $$D_{\mathrm{consistent}}={\mathrm{Invalidate}}_{\mathrm{PS}}\left(D_{\mathrm{PL}\text{\_}\mathrm{out}}\right)$$

Execution of Equation (12) invalidates the stale cache lines in the PS, ensuring that the subsequent processing stages fetch the deterministic results directly from the external DDR memory. Because the structural isomorphism of RS-YOLO enforces a fixed, predictable dataflow lifecycle, this explicit synchronization model avoids dynamic tracking overhead, securing data consistency with minimal power overhead.

#### 2.4.2. Overview of Acceleration Architecture

**Figure 6:**
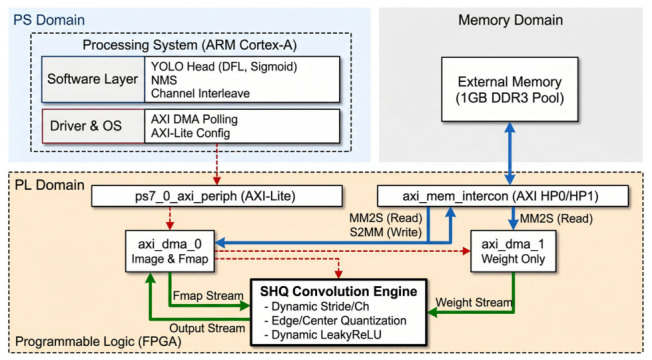
System architecture diagram, blue solid arrows denote AXI memory transfer paths, red dashed arrows indicate control paths, and green solid arrows represent internal FPGA streaming data paths.

The complete acceleration framework of RS-YOLO is physically instantiated on the Xilinx Zynq-7020 heterogeneous SoC platform, as illustrated in Figure 6. By tightly coupling the PS and PL domains, the architecture achieves a seamless integration between high-level algorithmic management and the underlying hardware execution layer.

At the system-level functional partitioning level, tasks are partitioned based on computational regularity:

- The PS domain coordinates the global FSM, schedules the system DMA controllers, and executes non-linear macro-tasks including bounding box decoding and non-maximum suppression (NMS).
- The PL domain hosts the custom high-performance SHQ engine, dedicating its internal logic fabric entirely to accelerating the throughput of the deterministic dataflow.

At the high-speed data transfer level, external DDR memory bandwidth utilization is maximized via dual independent AXI4-Stream channels. The feature stream pipeline routes feature maps through DMA controllers connected directly to the high-bandwidth HP ports. Simultaneously, a dedicated weight stream path pulls parameter blocks from the external DDR memory into the on-chip BRAM buffers, ensuring that the processing element (PE) array can respond instantaneously to inter-layer transitions.

To ensure the synchronized transmission of control parameters, the AXI4-Lite bus maps the hardware configuration registers, enabling the PS driver to dynamically update convolution strides, input and output channel dimensions, and the specific dequantization parameters required by the SHQ engine. This hardware–software co-design successfully decouples unpredictable control logic management from highly uniform data-parallel computations. While limiting the total system power consumption to only 2.24 W, the integration establishes a stable, high-efficiency pipeline optimized for real-time intelligent interpretation on resource-limited edge platforms.

## 3. Results

### 3.1. Experimental Setup

The NWPU VHR-10 dataset [31] contains 800 high-resolution remote sensing images, with image sizes ranging from approximately 533×597 to 1728×1028 pixels and a spatial resolution of roughly 0.08 to 2 m. Among these, 650 images contain annotated objects (comprising 3896 instances), while the remaining 150 are negative samples. We partitioned the positive samples into training, validation, and testing sets at a ratio of 8:1:1. Specifically, this yields 520 images for training, 65 for validation, and 65 for testing. To strictly eliminate the potential interference of weight initialization noise and guarantee the statistical generalizability of the proposed framework, all evaluated models were re-trained and cross-validated across five independent random seeds (including seeds 35, 15, 66, 24, and 2), and the experimental metrics are reported as mean±standard deviation (SD).

The experimental hardware platforms are divided into a training platform and a deployment platform. The training platform is equipped with an Intel(R) Core(TM) i5-10300H CPU @ 2.50 GHz and an NVIDIA GeForce GTX 1650 Ti GPU. The deployment platform utilizes the Xilinx Zynq-7020 FPGA. The software environment is built on Python 3.11.14, PyTorch 2.4.1, and CUDA 11.8. Hardware synthesis and implementation were accomplished using Vivado 2022.2 and Vitis HLS 2022.2. The training parameters were configured as follows: input image size of 512×512, initial learning rate of 0.01, batch size of 16, Stochastic Gradient Descent (SGD) optimizer, and 200 training epochs. Evaluation metrics include Precision, Recall, ${\mathrm{mAP}}_{50}$, and ${\mathrm{mAP}}_{50:95}$, alongside hardware-specific metrics such as resource utilization, power consumption, and energy efficiency. For the Zynq-7020 platform, total power consumption was analyzed using the Vivado 2022.2 tool. Throughput was calculated by multiplying the theoretical computational cost per frame by the number of frames processed.

### 3.2. Algorithm Performance and Ablation Analysis

To verify the effectiveness of the proposed RS-YOLO model at the algorithmic level, this paper conducted a comparative study against several mainstream lightweight object detectors (YOLOv5n, YOLOv8n, YOLOv9t, YOLOv10n, and YOLOv13n) under identical experimental settings. All models were evaluated on the NWPU VHR-10 dataset, with key performance metrics summarized in Table 1 and Table 2.

As shown in the experimental results, RS-YOLO achieves the highest detection accuracy among the compared models, with a ${\mathrm{mAP}}_{50}$ of 0.900 and a ${\mathrm{mAP}}_{50:95}$ of 0.591. Compared with YOLOv9t, these values represent improvements of 2.3% and 1.0%, respectively. In addition, RS-YOLO achieves a recall of 0.860, which is 1.4% higher than that of YOLOv8n. These results indicate that the proposed network architecture effectively improves the detection of dim and small targets while maintaining the advantages of a lightweight detector.

In terms of model complexity, RS-YOLO contains only 1.11 M parameters, representing a 63.1% reduction compared with YOLOv8n (3.01 M). This reduction is accompanied by a moderate increase in theoretical computational complexity. Compared with YOLOv8n, RS-YOLO reduces the computational complexity from 8.1 GFLOPs to 7.34 GFLOPs while achieving higher detection accuracy.

To further quantify the contribution of each proposed component, an ablation study was conducted under the same experimental conditions, as summarized in Table 1. Starting from the baseline YOLOv8n model, which achieves a ${\mathrm{mAP}}_{50}$ of 0.882, introducing the RepStraight module alone increases the ${\mathrm{mAP}}_{50}$ to 0.892 and improves the recall from 0.846 to 0.870, while simultaneously reducing the computational complexity from 8.1 GFLOPs to 6.6 GFLOPs. These results indicate that the proposed structural re-parameterization not only simplifies the network topology for deterministic hardware execution but also improves feature representation. Introducing the P2 feature branch alone further increases the ${\mathrm{mAP}}_{50}$ to 0.894 while reducing the number of parameters to 2.54 M. This improvement demonstrates the effectiveness of high-resolution feature representations for detecting dim and small targets. Crucially, when both components are synergistically integrated into the complete RS-YOLO framework, an orthogonal optimization dividend is unlocked. The proposed model achieves a peak. When the RepStraight module and the P2 feature branch are integrated into the complete RS-YOLO framework, the model achieves an ${\mathrm{mAP}}_{50}$ of 0.900 and a recall of 0.860, while reducing the parameter count to 1.11 M. These results indicate that the two components provide complementary benefits. The RepStraight module simplifies the network topology for hardware implementation, whereas the P2 feature branch improves the representation of fine-grained spatial information. Their combination enables RS-YOLO to achieve improved detection accuracy while maintaining a compact model suitable for resource-constrained edge platforms.

### 3.3. Architectural Ablation on Hardware Efficiency

To quantitatively evaluate the hardware benefits introduced by the proposed RepStraight module, an architectural ablation study was conducted on the Xilinx Zynq-7020 platform. Two hardware implementations were synthesized under identical operating conditions: a baseline implementation based on the original YOLOv8n architecture with standard C2f modules and the proposed RS-YOLO architecture incorporating RepStraight. To ensure a fair comparison, both implementations employed the same 8-bit fixed-point quantization scheme and operated at an identical clock frequency. The corresponding hardware resource utilization and timing results are summarized in Table 3.

The synthesis results show that the proposed structural re-parameterization substantially reduces hardware resource consumption. By removing the parallel branches, channel-splitting operations, and feature concatenation paths in the original C2f module, RepStraight eliminates the branch-selection control signals and associated data-path multiplexers required for switching between 1 × 1 and 3 × 3 convolution branches. RTL-level analysis further shows that the overall FSM scheduling framework remains unchanged in both implementations. The state-transition graph and its encoding are preserved, while the combinational control logic within each state is simplified. In particular, the branch arbitration logic and inter-branch synchronization circuitry required by the heterogeneous computation graph are eliminated. As a result, LUT utilization is reduced by 24.7%, decreasing from 18.2 k to 13.7 k, while distributed LUTRAM utilization decreases by 43.7%, from 921 to 518. These improvements reflect the reduced routing and control complexity introduced by the proposed structural regularization.

In addition to reducing hardware resource consumption, RepStraight transforms the heterogeneous computation graph into a deterministic single-path dataflow. Without branch synchronization or runtime switching between heterogeneous convolution operators, the hardware pipeline executes continuously without waiting for intermediate feature synchronization. Consequently, the end-to-end inference latency on the Zynq-7020 platform is reduced from 341.2 ms to 225.4 ms, corresponding to a 33.9% reduction. The resulting implementation achieves a throughput of 4.44 FPS. These results indicate that simplifying the network topology effectively reduces hardware control overhead and improves execution efficiency under stringent SWaP constraints.

### 3.4. Quantization Accuracy Comparison

**Figure 7:**
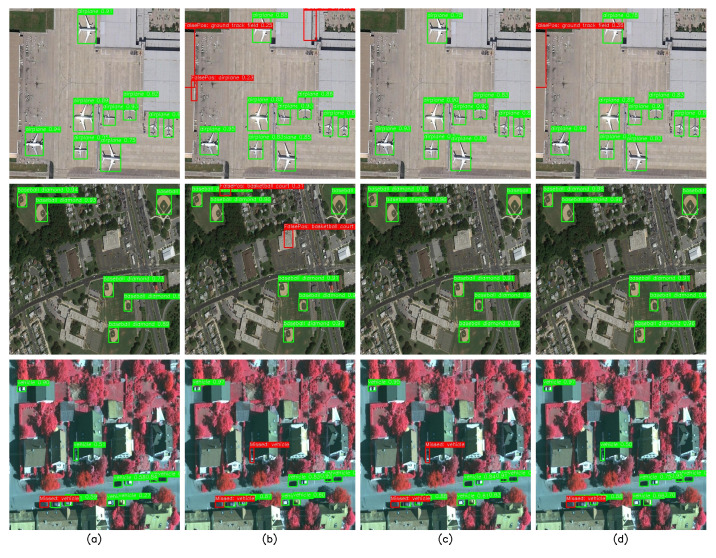
The detection results of RS-YOLO on the NWPU VHR-10 dataset, (**a**) is the baseline model, (**b**) is the INT8 model, (**c**) is the INT16 model, and (**d**) is the SHQ model, green indicates correct detection, while red indicates false detection or missed detection.

To evaluate the robustness of the SHQ scheme against quantization noise, an accuracy ablation study is conducted under varied bit-width constraints. The floating-point FP32 model executing on the host PC serves as the performance baseline, achieving a ${\mathrm{mAP}}_{50}$ of 0.901 on the NWPU VHR-10 test set. The quantized accuracy results are summarized in Table 4, with qualitative target localization profiles mapped in Figure 7.

When the network is quantized using a conventional uniform INT8 scheme, the detection accuracy decreases to a ${\mathrm{mAP}}_{50}$ of 0.872 and a ${\mathrm{mAP}}_{50:95}$ of 0.521. As discussed in Section 2.2, this performance degradation is primarily attributed to the spatial energy centralization introduced by structural re-parameterization. Because the central coefficient of the fused convolution kernel exhibits a substantially larger dynamic range than the surrounding coefficients, uniform quantization enlarges the global quantization step size, thereby reducing the representation precision of low-energy peripheral coefficients.

Increasing the quantization precision to uniform INT16 partially recovers the accuracy, improving the ${\mathrm{mAP}}_{50}$ to 0.891 and ${\mathrm{mAP}}_{50:95}$ to 0.547. However, the increased bit width also leads to higher hardware cost, including greater DSP utilization and routing complexity, resulting in an end-to-end inference latency of 492.8 ms, which is more than twice that of the uniform INT8 implementation.

By contrast, the proposed SHQ scheme achieves a ${\mathrm{mAP}}_{50}$ of 0.887 and a ${\mathrm{mAP}}_{50:95}$ of 0.553. Although its ${\mathrm{mAP}}_{50}$ remains slightly lower than that of the uniform INT16 implementation, the SHQ scheme achieves a higher ${\mathrm{mAP}}_{50:95}$, improving the localization accuracy by 0.6 percentage points. These results indicate that selectively preserving higher precision for the high-entropy central coefficient is more effective than uniformly increasing the quantization precision across the entire kernel.

From the hardware perspective, the dedicated 16-bit computation path and delayed dequantization alignment strategy enable the SHQ engine to limit the inference latency to 242.7 ms. Compared with the uniform INT8 implementation, this introduces only a 7.7% latency increase, while reducing the latency by 50.7% relative to the uniform INT16 implementation. These results demonstrate that the proposed SHQ scheme effectively balances quantization accuracy and hardware efficiency, making it well suited for resource-constrained FPGA-based edge inference.

### 3.5. Hardware Performance Evaluation

#### 3.5.1. Resource Utilization Analysis

The customized spatial heterogeneous acceleration engine is implemented using High-Level Synthesis tools and fully deployed on the physical Xilinx Zynq-7020 silicon fabric. Driven by a stable hardware clock frequency of 100 MHz, the synthesis utilization metrics are systematically detailed in Table 5.

It should be noted that Table 5 reports the post-implementation resource utilization of the complete RS-YOLO system with the SHQ engine enabled. The increase in DSP utilization from 189 (Table 2, where both YOLOv8n and RS-YOLO are evaluated under uniform INT8 quantization for structural comparison) to 220 is attributed to the SHQ design, which instantiates two parallel arithmetic pathways: a vectorized 8-bit packing pipeline for peripheral coefficients and an independent 16-bit multiplication pathway for central coefficients (Section 2.3). Although this dual-path structure increases DSP consumption, it enables heterogeneous precision computation without increasing the global bit-width. The LUT increase from 13.7 k to 18.6 k is mainly caused by the cross-scale dequantization alignment logic and the ap_uint<100> wide-word weight storage format required for heterogeneous coefficient packing.

The post-implementation synthesis results indicate that the system exhibits a compute-dominant resource profile consistent with the structural isomorphism design. In particular, DSP48E utilization reaches full occupancy (100%), indicating that the computational pipeline effectively saturates the available multiply–accumulate resources of the FPGA fabric. This suggests that most arithmetic operations are mapped onto the systolic execution array with minimal idle compute capacity.

Meanwhile, LUT utilization remains at 35%, reflecting reduced combinational control complexity due to the RepStraight structure, which eliminates heterogeneous branch selection and routing logic. In addition, the use of ap_uint<100> wide-word storage leads to low utilization of distributed LUTRAM and FF resources (4% and 16%, respectively), as most data movement is handled in a streaming-oriented manner rather than fine-grained control logic.

The BRAM utilization is measured at 55%, which supports the line-buffer and feature-map storage requirements of the high-resolution P2 pathway. This configuration indicates that the architecture shifts resource usage from control-oriented logic toward structured data buffering and streaming computation, improving execution predictability under resource-constrained FPGA conditions.

From a system-level perspective, the proposed accelerator exhibits a compute-dominant behavior on the Zynq-7020 platform, where DSP48E slices reach full utilization (100%). This indicates that the execution pipeline is primarily constrained by arithmetic throughput rather than memory bandwidth under resource-limited deployment conditions. Meanwhile, moderate LUT utilization (35%) reflects reduced combinational control complexity enabled by the RepStraight structure, which eliminates heterogeneous branch selection and routing logic. In addition, BRAM utilization (55%) is sufficient to support on-chip buffering and feature-map storage without becoming the primary performance bottleneck. Overall, the streaming-oriented execution pattern reduces memory access contention, and system performance on the Zynq-7020 platform is mainly limited by available computational resources rather than data movement or memory hierarchy constraints.

#### 3.5.2. Comparison with Existing FPGA Accelerators

To evaluate the performance of the proposed co-designed architecture, a cross-benchmark comparison is executed against mainstream accelerators from the recent literature. The performance comparisons are summarized in Table 6.

When evaluated on the Zynq-7020 platform at 100 MHz, the proposed design requires 18.6 k LUTs, corresponding to a 60.3% reduction compared with the 46.9 k LUTs reported in Fang et al. The total power consumption is measured at 2.24 W, achieving an energy efficiency of 15.19 GOPS/W, which is approximately 4.17× higher than the 3.64 GOPS/W reported in Fang et al.

In terms of throughput–efficiency trade-offs, the accelerator proposed by Babu et al. (Zynq-7020) reports 18.25 GOPS/W, while the design by Yan et al. (ZCU102) achieves 65.7 GOPS/W. However, these implementations operate at higher power budgets of 10.36 W and 12.3 W, respectively. In comparison, the proposed framework maintains a significantly lower power consumption of 2.24 W on Zynq-7020 and 6.96 W on ZCU102, while preserving a quantized detection accuracy of 0.887 mAP50.

Although the compared methods may achieve higher peak efficiency or throughput under their respective configurations, these differences primarily arise from inconsistencies in experimental settings, including variations in FPGA devices, network architectures, operating frequencies, input resolutions, and quantization strategies (e.g., YOLOv4, YOLOv5s, YOLOv8n). In particular, hardware platforms differ significantly in available DSP resources, memory hierarchies, and routing architectures, which directly affect achievable throughput and energy efficiency. Therefore, the reported results should be interpreted as system-level performance under specific deployment conditions rather than strictly comparable indicators of absolute architectural superiority. The comparison is intended to highlight overall efficiency trends of hardware–algorithm co-design instead of claiming absolute optimality across heterogeneous implementations.

## 4. Discussion

### 4.1. Synergistic Mechanism of Structural Isomorphism and SHQ

The performance improvements achieved by RS-YOLO in terms of hardware efficiency and detection accuracy are primarily attributed to the coordinated design of structural re-parameterization (RepStraight) and the SHQ strategy. Existing methods often treat network simplification and quantization as separate optimization procedures, which may lead to mismatches when deployed on FPGA-based architectures.

In the proposed framework, the RepStraight module transforms the original multi-branch topology into a uniform sequential structure composed of 3 × 3 convolutions prior to deployment. This transformation removes intra-layer branching operations and short-range residual paths, resulting in a simplified and deterministic execution graph. By eliminating intermediate feature buffering and branch synchronization requirements, the memory access pattern becomes more regular, and the combinational control logic associated with dynamic routing is significantly reduced. Consequently, the computation can be executed as a single-path dataflow, improving the utilization efficiency of DSP-based processing elements under continuous streaming conditions.

The SHQ engine is designed to address the non-uniform coefficient distribution introduced by structural re-parameterization. After fusion of multi-branch operators, the central kernel coefficient aggregates contributions from multiple computational paths, resulting in a higher dynamic range compared with peripheral elements.

To mitigate accuracy degradation under uniform quantization, a spatially differentiated bit-width strategy is adopted. A dedicated 16-bit multiplication path is assigned to the central coefficient, while peripheral coefficients are processed using an 8-bit pipeline. This design preserves fine-grained feature representation, particularly for low-contrast and small targets, while maintaining hardware efficiency under resource-constrained conditions.

Although the proposed method is evaluated on the NWPU VHR-10 dataset, which is relatively small compared with large-scale benchmarks such as DOTA or DIOR, the primary goal of this study is not to maximize benchmark diversity but to validate the effectiveness of the hardware-software co-design framework under tightly constrained FPGA-based edge environments. In this context, NWPU VHR-10 provides a representative set of high-resolution aerial scenes with significant intra-class variation and small-target characteristics, making it suitable for evaluating both detection robustness and hardware-aware optimization behavior under realistic spaceborne deployment constraints.

Overall, the results indicate that the combination of structural isomorphism and spatially adaptive quantization provides a practical mechanism for improving the trade-off between computational determinism and inference accuracy in FPGA-based edge inference systems.

### 4.2. Hardware Boundary Limitations and Spaceborne Throughput Bottlenecks

Although the proposed architecture improves execution efficiency under SWaP-constrained conditions, its performance remains bounded by hardware resource scale and fixed deployment configuration.

The implementation operates at 100 MHz on the Xilinx Zynq-7020 platform to satisfy a total power budget of 2.24 W. While RepStraight improves structural regularity and increases DSP utilization efficiency, the fixed operating frequency inherently limits overall throughput scalability.

The resulting inference latency is 242.7 ms. This level of performance is suitable for low-power edge inference tasks with moderate temporal requirements, but may not fully meet high-frame-rate continuous monitoring scenarios.

In addition, the SHQ mechanism relies on a static quantization configuration determined offline. Although this design improves hardware determinism, it reduces adaptability under varying input distributions, such as changes in illumination conditions, sensor characteristics, or scene-dependent dynamic ranges. Under such conditions, fixed scaling parameters may lead to sub-optimal reconstruction of low-contrast features.

These observations suggest that further improvements are required in adaptive precision control and runtime-aware scheduling mechanisms.

### 4.3. Future Extensions for Multi-Spectral Spaceborne Payloads

Future work will focus on enhancing the adaptability and robustness of the proposed architecture in more dynamic and heterogeneous deployment environments.

A first direction is the exploration of runtime adaptive quantization mechanisms. Instead of relying on fixed SHQ parameters, future designs may incorporate lightweight statistical estimation modules capable of analyzing feature distribution characteristics online. Based on these estimates, bit-width allocation and scaling factors can be dynamically adjusted to better accommodate variations in scene complexity and sensor conditions.

A second direction is the investigation of fault-resilient computation strategies for long-duration deployment scenarios. In practical edge environments, transient faults, numerical perturbations, and aging effects may impact computational reliability. Introducing redundancy-aware computation or error-mitigation mechanisms within the systolic dataflow could improve robustness without significantly increasing hardware overhead.

A third direction is the extension toward multi-modal and multi-spectral inference tasks. Current design focuses on single-stream optical imagery, while future remote sensing systems increasingly rely on fused information from multiple spectral bands. Extending the proposed structural isomorphism and SHQ framework to heterogeneous input modalities may further improve its applicability in complex observation scenarios.

## 5. Conclusions

This work presents RS-YOLO, a hardware-software co-designed object detection framework targeting efficient inference under resource-constrained edge conditions. The proposed architecture integrates structural re-parameterization (RepStraight) and spatial heterogeneous quantization (SHQ) to jointly improve computational regularity and numerical robustness on FPGA platforms.

Experimental results on the NWPU VHR-10 dataset and implementation on the Xilinx Zynq-7020 platform demonstrate that the proposed model achieves a compact parameter size of 1.11 M and a quantized mAP of 0.887. Compared with uniform 8-bit quantization, the proposed SHQ strategy improves localization performance, particularly for low-contrast and small targets, under the same hardware constraints.

From a system perspective, the elimination of multi-branch computation patterns reduces control-path complexity and improves dataflow determinism, resulting in a total power consumption of 2.24 W and an energy efficiency of 15.19 GOPS/W. These results indicate that aligning network topology with hardware execution structure is an effective strategy for improving FPGA-based edge inference efficiency.

It should be emphasized that the current work focuses on FPGA-based validation under controlled experimental conditions. Further investigation is required to evaluate long-term reliability and robustness under more challenging operational environments, including radiation effects and sensor variability.

## Figures and Tables

**Table 1:** Algorithm performance comparison.

Models	${\mathrm{mAP}}_{50}$	${\mathrm{mAP}}_{50:95}$	Precision	Recall	Parameters	GFLOPs
YOLOv5n	0.863 ± 0.012	0.545 ± 0.013	0.843 ± 0.017	0.834 ± 0.008	2.5	7.1
YOLOv8n	0.882 ± 0.007	0.553 ± 0.019	0.881 ± 0.026	0.846 ± 0.006	3.01	8.1
YOLOv9t	0.877 ± 0.009	0.581 ± 0.008	0.891 ± 0.013	0.838 ± 0.008	2.01	7.6
YOLOv10n	0.87 ± 0.011	0.572 ± 0.015	0.836 ± 0.015	0.842 ± 0.010	2.26	6.5
YOLOv13n	0.854 ± 0.014	0.539 ± 0.021	0.833 ± 0.019	0.822 ± 0.015	2.37	5.2
RS-YOLO	0.900 ± 0.007	0.591 ± 0.009	0.895 ± 0.010	0.860 ± 0.014	1.11	7.34

**Table 2:** Ablation Analysis.

Models	RepStraight	P2	${\mathrm{mAP}}_{50}$	${\mathrm{mAP}}_{50:95}$	Precision	Recall	Parameters	GFLOPs
YOLOv8n	×	×	0.882 ± 0.007	0.553 ± 0.019	0.881 ± 0.026	0.846 ± 0.006	3.01	8.1
+RepStraight	√	×	0.892 ± 0.009	0.573 ± 0.012	0.891 ± 0.008	0.870 ± 0.013	4.00	6.6
+P2	×	√	0.894 ± 0.005	0.585 ± 0.015	0.901 ± 0.011	0.845 ± 0.007	2.54	7.78
RS-YOLO	√	√	0.900 ± 0.007	0.591 ± 0.009	0.895 ± 0.010	0.860 ± 0.014	1.11	7.34

Note: √ indicates that the corresponding component is enabled, whereas × indicates that the corresponding component is not included.

**Table 3:** Architectural ablation on hardware efficiency.

Models	LUT	LUTRAM	FF	BRAM	DSP	Latency [ms]
YOLOv8n	18.2 k	921	16.9 k	66.5	189	341.2
RS-YOLO	13.7 k	518	14.7 k	66.5	189	225.4

**Table 4:** Comparison of Quantization Accuracy.

Device	Precision	$mAP_{50}$	$mAP_{50:95}$	Latency [ms]
PC	FP32	0.900	0.591	78.5
Zynq 7020	INT8	0.872	0.521	225.4
Zynq 7020	INT16	0.891	0.547	492.8
Zynq 7020	SHQ	0.887	0.553	242.7

**Table 5:** Resource utilization on Zynq-7020.

Resource	LUT	LUTRAM	FF	BRAM	DSP
Utilization	18,658	759	17,371	76.50	220
Available	53,200	17,400	106,400	140	220
Utilization	35%	4%	16%	55%	100%

**Table 6:** Comparison with existing accelerators.

Metric	Angel-Eye [32]	Fang et al. [17]	Zhang et al. [15]	Afzal et al. [33]	Babu et al. [16]	Yan et al. [18]	Ours	
Year	2018	2025	2020	2020	2022	2024	2026	2026
device	Z045	Z7020	ZCU102	VC707	Z7020	ZCU102	Z7020	ZCU102
LUTs	182 k	46.9 k	95 k	198 k	23.2 k	160 k	18.6 k	74.7 k
FFs	127 k	40.9 k	90 k	117 k	45.8 k	N/A	17.3 k	10.9 k
BRAMs	486	107	491	1027	115	558	76.5	649
DSPs	780	205	609	1926	174	1647	220	1433
Power [W]	9.63	2.8	11.8	9.26	10.36	12.3	2.24	6.96
Frequency [MHz]	150	100	300	200	100	200	100	150
Precision	16-bit	N/A	16-bit	18-bit	N/A	4-bit	SHQ	SHQ
GOPs/W	14.2	3.64	8.64	11.65	18.25	65.7	15.19	39.35

## Data Availability

The data used in this study are publicly available datasets, namely NWPU VHR-10 (https://www.kaggle.com/datasets/larbisck/nwpu-vhr-10 (accessed on 8 October 2025)).

## Abbreviations

The following abbreviations are used in this manuscript:

SWaP	Size, Weight, and Power
YOLO	You Only Look Once
CNN	Convolutional Neural Network
FPGA	Field-Programmable Gate Array
HLS	High-Level Synthesis
DSP	Digital Signal Processor
BRAM	Block Random Access Memory
FSM	Finite State Machine
LUT	Look-Up Table
FF	Flip-Flop
SHQ	Spatial Heterogeneous Quantization
INT8	8-bit Integer
INT16	16-bit Integer
PS	Processing System
PL	Programmable Logic
DMA	Direct Memory Access
DDR	Double Data Rate
HP	High Performance
MAC	Multiply–Accumulate
BN	Batch Normalization
SPPF	Spatial Pyramid Pooling Fast
FPN	Feature Pyramid Network
PAN	Path Aggregation Network
mAP	mean Average Precision
GOPs	Giga Operations per second
GFLOPs	Giga Floating Point Operations
PTQ	Post-Training Quantization
RTL	Register Transfer Level
SoC	System on Chip
AXI	Advanced Extensible Interface
